# External prescription circulation and chronic medication refills via internet hospital: a real-world study of patient characteristics in eastern China

**DOI:** 10.3389/fpubh.2026.1855582

**Published:** 2026-08-27

**Authors:** Ying Zhang, Zhe Feng, En-feng Li, Jinqiu Hu

**Affiliations:** Hospital Administrative Office, Xishan People’s Hospital of Wuxi City, Wuxi, China

**Keywords:** chronic medication prescriptions, digital health equity, external prescription, internet hospital, patient characteristics, real-world study

## Abstract

**Background:**

The COVID-19 pandemic has accelerated the adoption of internet-based care and electronic prescription systems, yet real-world evidence describing patient characteristics and usage patterns for hospital-led external prescription circulation and chronic medication refill services remains limited. Such evidence is urgently needed to guide service optimization and health equity in digital chronic disease management.

**Methods:**

A single-center, retrospective, real-world study was conducted at a district-level hospital in eastern China. Data were collected over 65 days (January 23 to March 28, 2026), including 17,528 online chronic medication refill visits and 172,232 offline outpatient encounters. Patient demographics, temporal usage patterns, and prescription profiles were analyzed. Group comparisons were performed using chi-square tests, independent-samples *t*-tests, and effect-size statistics. A multivariable Generalized Estimating Equation (GEE) model was performed to identify independent predictors of evening service utilization. To quantify the macro-level impact of the Spring Festival on daily prescription volume, we additionally performed a negative binomial interrupted time series analysis adjusting for day-of-week and secular trend.

**Results:**

The online patient cohort was characterized by an older demographic (mean age 55.0 years), with 84.85% aged ≥40 years. Distinct stratification was observed internally: older patients favored morning usage (08:00–10:00), whereas younger patients peaked in the evening (18:00–20:00; Cramér’s *V* = 0.165, *p* < 0.001). Cardiovascular drugs predominated among older patients, while younger patients used more Chinese patent medicines, digestive agents, and anti-inflammatory drugs (Cramér’s *V* = 0.167, *p* < 0.001). Multivariable GEE analysis identified older age (≥40 years) (Wald *χ*^2^ = 158.787, *p* < 0.001), male gender (Wald *χ*^2^ = 10.845, *p* = 0.001), the Spring Festival period (Wald *χ*^2^ = 23.346, *p* < 0.001), and chronic disease type (Wald *χ*^2^ = 6.041, *p* = 0.014) as independent predictors for evening use.

**Conclusion:**

Hospital-based internet prescription refill services effectively target middle-aged and older patients with chronic conditions and demonstrate strong age-related differences in timing and medication needs. These findings support stratified, user-centered digital health design rather than one-size-fits-all models. Wider implementation can improve equitable access to chronic medications, optimize resource allocation, and strengthen chronic disease care in the digital health era.

## 1 Introduction

The 2019 novel coronavirus disease (COVID-19) global pandemic has significantly transformed the organization and provision of worldwide health care. It has sped up the adoption of telehealth, electronic medication prescribing, and internet-based healthcare models. These are ways to maintain the continuity of care while reducing exposure to infectious hazards (1–4). For people who have chronic conditions that need continuous medication refills, such as high blood pressure, diabetes, heart-related problems, and long-lasting pain, having continuous access to prescription treatments is crucial to stop clinical decline, prevent unnecessary hospital stays, and avoid negative results (5). Yet traditional outpatient pharmacy services are still restricted by extended waiting periods, restricted in-person availability, and poor coverage for geographically scattered or medically at-risk groups (6). These persistent disparities highlight an evident and pressing requirement for scalable, patient-focused models that combine in-person hospital treatment with efficient digital prescription services.

In this context, the combination of internet hospital platforms and physical hospital systems has become a practical approach to enhance chronic disease management and modernize the medication supply chain. Internet medical institutions enable remote consultations, issue electronic prescriptions, and share prescriptions across different institutions. This way, patients can get repeat medication refills without the need for repeated in-person consultations. Of specific significance, external prescription distribution and long-term medication refill services connect hospitals with partnered retail pharmacies. This allows for smooth electronic transfer, real-time dispensing, and greater convenience for patients. These elements together improve medication availability and compliance (6). This comprehensive model is in line with worldwide trends regarding value-based healthcare, digital health evolution, and patient-centered service delivery (1).

Despite the increasing adoption, solid evidence is still scarce regarding the real-world performance, patient usage patterns, and clinical effects of external refill services provided by physical hospitals via internet-based platforms (1, 6). Most current research efforts concentrate on the adoption of telehealth, the precision of e-prescriptions, or the quality of dispensing in primary healthcare or community contexts. Relatively less focus has been given to the hybrid approach that integrates physical hospitals with internet-based prescription processes (6). Although multiple reports indicate an increase in telehealth utilization, a decrease in in-person consultations, and more frequent electronic prescribing during the pandemic (2, 3), only a small number have methodically investigated age-related differences, temporal usage trends, or drug class distributions in chronic disease refill services (1). Furthermore, even when guidelines have been formulated to aid in safe tele-prescribing during public-health emergencies, high-quality real-world data that compare patient subgroups within this integrated care framework are still scarce (1, 6).

The objective of the present study is to describe patient characteristics, time-related usage trends, and prescription details linked to external prescription dissemination and long-term medication refill services provided through an internet hospital platform run by a district-level general hospital. Age-associated differences in service utilization and medication types are examined to recognize disparities and actionable trends. The results are meant to back evidence-based enhancements of combined internet-physical hospital services, reinforce chronic disease control, provide information for health policies, and promote fair access to necessary medications for at-risk populations (1).

The growth of internet hospital services and the implementation of national medical insurance electronic prescription circulation are crucial elements of China’s healthcare system reform and the coordinated progress of medical treatment, health insurance, and pharmaceutical services—the three medical linkages. Since 2018, the National Healthcare Security Administration (NHSA) of China has rolled out a set of policies to establish a comprehensive regulatory system.

In 2018, official pricing criteria were set for Internet Plus medical services. In 2021, the two-channel management mechanism was put into effect for nationally negotiated drugs covered by insurance. A significant expansion took place in 2023. At that time, the nominated retail pharmacies were incorporated into the outpatient pooled reimbursement plan. This expanded the scope of electronic prescription coverage from just a limited group of dual-channel drugs to almost all outpatient pharmaceutical services. In October 2024, the Notice on Regulating the Management of External Prescription Dispensing for Medical Insurance Drugs further outlined requirements: commencing on January 1, 2025, all dual-channel medications should circulate solely via the national healthcare security electronic prescription center, and cross-region external prescriptions are typically restricted.

At the provincial-level, local governments in Hubei, Jiangsu, Jiangxi and other areas have formulated phased implementation schemes according to local circumstances. These endeavors depend on the unified national healthcare security information platform to standardize the deployment of electronic prescription centers. The regulatory system requires RSA-AES hybrid encryption for data safety, enhanced intelligent prescription assessment, and whole-chain supervision to prevent fraud and improper use.

Throughout China, health authorities keep on perfecting implementation via workflow streamlining and formal cooperation between chain drugstores and internet hospitals. These measures are intended to regularize the circulation of prescriptions, guarantee the safe utilization of insurance funds, and enhance patients’ access. Collectively, these policies have established a distinct institutional and operational route for physical hospitals in China to advance internet hospital services and expand insurance-related electronic prescription circulation.

Backed by the national healthcare security information platform and the extensive application of medical insurance electronic vouchers, the technical foundation for electronic prescription circulation has grown more mature and dependable. In November 2019, the NHSA rolled out medical insurance electronic vouchers in Jinan (Shandong province), setting up a unified national standard for integrating insurance services with internet-based medical care. As the distinctive digital identifier for the insured, these vouchers allow for smooth identity verification, online settlement, and prescription sharing among platforms.

Technically, well-developed electronic prescription systems usually adopt a platform-plus-interface architecture. This architecture enables interoperability among hospital information systems (HIS), healthcare security platforms, and pharmacy settlement systems via standardized application programming interfaces (APIs). Key technological protections involve digital signatures to guarantee prescription integrity and non-repudiation, RSA-AES hybrid encryption for safeguarding data transmission, and smart review systems that evaluate prescribing rationality based on patient characteristics, diagnoses, and prescription particulars. Blockchain-empowered traceability and big-data-centered insurance fund supervision further strengthen system safety, steadiness, and responsibility.

Today, the insurance e-prescription circulation system acts as the key digital avenue for external prescription issuance and insurance payment in internet hospital services. Meanwhile, internet hospitals are the main clinical settings for prescription circulation. These two systems are highly integrated in technological standards, infrastructure, work processes, and regulatory supervision, jointly establishing the fundamental digital framework for Internet Plus Health Care in China.

## 2 Methods

### 2.1 System architecture

In line with the technical specifications released by the National Healthcare Security Administration, a district-level general hospital in the eastern part of China built a practical system framework named Internet, Hospital Three Terminals and One Platform. This architectural design aimed to achieve complete compatibility with the hospitals previously established information infrastructure and operational work-processes (7).

Internet Plus Hospital Terminal: Functioning as the main hub for generating prescriptions, this terminal combines online follow-up consultations, asynchronous text-and-image consultations, pharmacist review services, and online pharmacy dispensing. Doctors communicate with patients from a distance to record clinical histories, assess reported complaints, and produce standardized electronic prescriptions.

Physician Workstation: This clinical module assists clinicians in creating prescriptions, conducting digital authorization, verifying electronic signatures, and securely uploading prescriptions to the regional healthcare platform.

Pharmacist Assessment Terminal: After a prescription is issued, pharmacists utilize this specialized interface to carry out prospective assessments of clinical suitability. Evaluations incorporate patient demographics, recorded indications, and prescription particulars to enhance medication safety and rationality.

Patient Care Portal: The portal designed for patients offers online consultation support, inquiries about prescription status, and real-time notifications regarding dispensing. Services are mainly provided through the hospital official WeChat account, and identity verification is carried out via electronic medical insurance credentials.

Medical Insurance System: This administrative system manages provincial and municipal drug formularies, hospital rosters, and contracted pharmacy inventories. It enables the activation of e-insurance vouchers, registration of beneficiaries, secure verification of e-prescriptions, and automatic gathering of settlement-relevant data.

### 2.2 Process design

**Figure 1:**
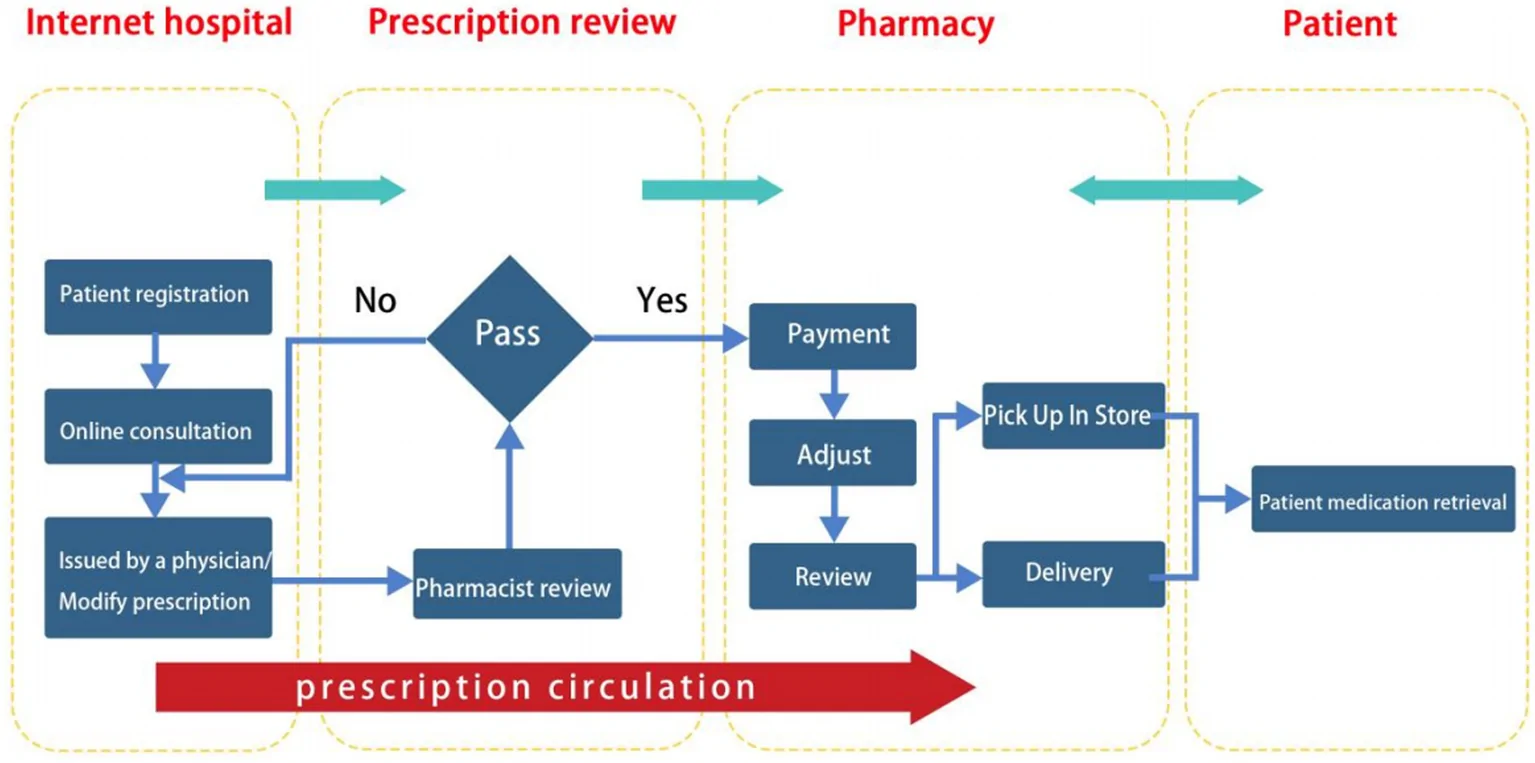
Workflow of the integrated internet hospital-based external prescription circulation and chronic medication refill service.

The workflow of medical insurance electronic prescription circulation was organized based on the Internet medical insurance electronic credential model. The complete operational route included patient identity verification, online consultation and applications for chronic disease prescription renewals, anticipated prescription scrutiny, electronic prescription transmittal, and pharmacy-based medicine dispensing, as depicted in Figure 1.

Identity Verification: Patients carried out real-name authentication by entering the Diagnosis and Treatment Services part through the hospital’s WeChat official account, adhering to standard processes to verify their identity.

**Figure 2:**
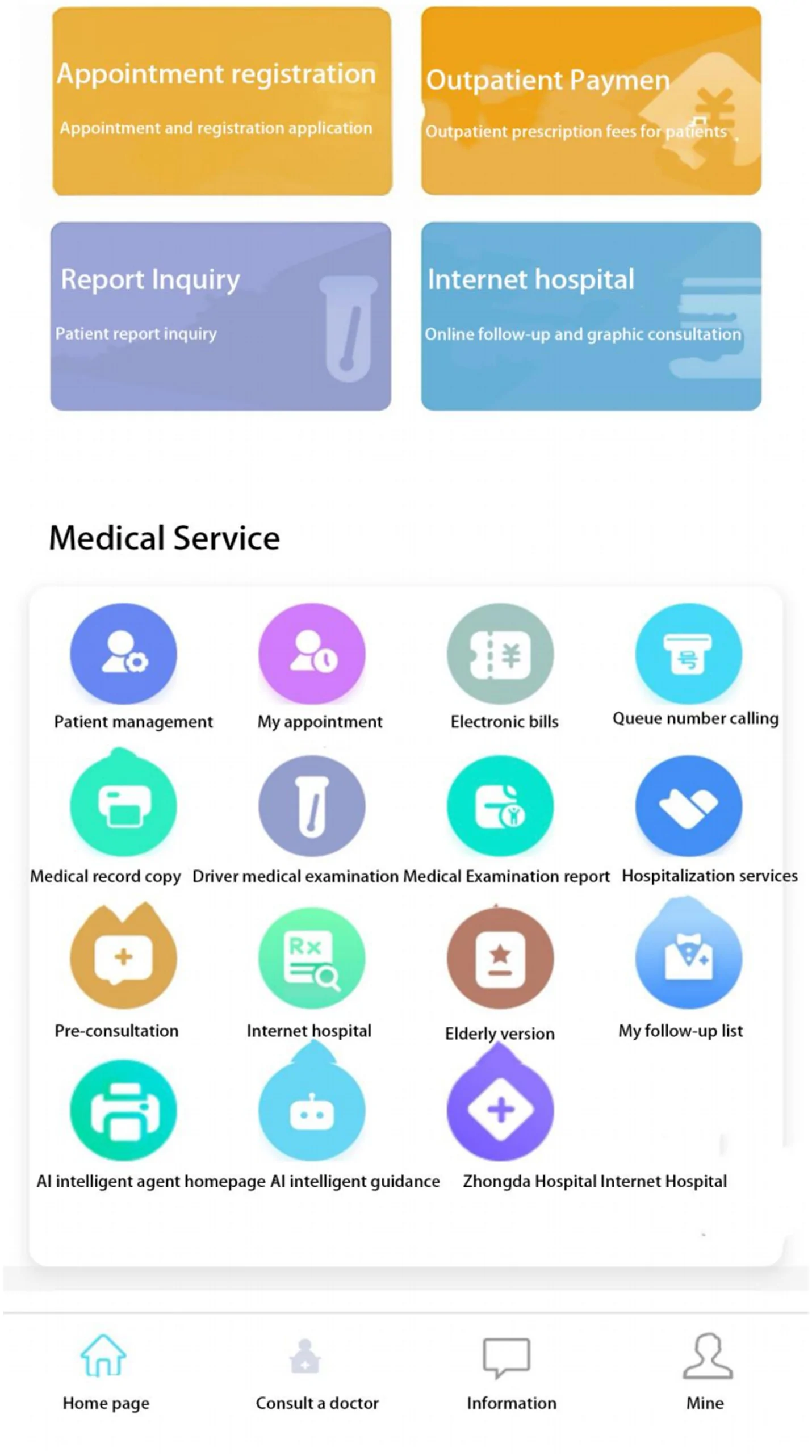
User interface of the internet hospital module on the hospital’s WeChat official account.

Online Consultation and Prescription Re-issue Request: Patients entered the internet hospital module via the hospital’s WeChat official account and chose the pharmacy service path to submit appointment and chronic disease medication refill requests, as presented in Figure 2. These requests included recorded diagnosis, drug specifications, and dosage details. Once the online settlement of consultation service fees via medical insurance was completed, the requests were directed to on-duty doctors for examination. Doctors then handled the applications and provided electronic prescriptions via the clinical workstation; when it was clinically required, they interacted with patients through text or image-based communications.

Prescription Assessment: Once prescriptions were issued, licensed pharmacists carried out prospective clinical evaluations. Each prescription was evaluated for rationality by integrating patient features, diagnostic data, and prescription particulars to guarantee clinical suitability and medication security.

Prescription Distribution: After passing the review and getting authorization successfully, prescriptions were uploaded to the regional medical insurance platform. Patients could later access valid prescription records via the Jiangsu Medical Insurance Cloud application.

In-Pharmacy Medicine Collection: Patients retrieved their medications at the specified contracted pharmacies within three calendar days following prescription approval. Identity authentication and medical insurance settlement were carried out by using the electronic medical insurance certificate or facial identification. Pharmacists provided medications solely after verifying eligibility and the authenticity of the final prescription.

### 2.3 Quality control

A comprehensive quality control framework covering pre-event, during-event, and post-event was set up to guarantee the safety, effectiveness, and compliance of the circulation of medical insurance electronic prescriptions in internet hospitals.

#### 2.3.1 Pre-event quality assurance

Physician Credential Supervision: A filing mechanism for prescription-accountable physicians was put in place. Doctors have to finish the hospital application, undergo pharmacy review, and obtain confirmation from the diagnosing hospital prior to getting registered in the medical insurance information system. Physicians who prescribe in internet hospitals must possess valid practicing credentials, finish regular training and evaluations, and uphold standardized medical practice. Following training and evaluation, 23 doctors with a general practice scope were approved as internet hospital prescribing physicians.

Drug Formulary Governance: A governance system for hospital external drug formularies was set up, where the Pharmacy and Therapeutics Committee conducts regular assessments of formulary inclusion and exclusion.

System Safety Control: A Class III cyber-security safeguarding system was employed, featuring multi-tiered tactics for user verification, data wholeness, and operational dependability.

Concurrent Quality Assurance: Dual Prescription Assessment Mechanism: A computerized clinical decision support-manual pharmacist review double-verification system was put into effect. The system conducts an automatic evaluation of indications, dosage, and drug–drug interactions through the integration of diagnosis, allergy history, and laboratory data. Prescriptions that do not pass automated screening are sent to pharmacists for manual assessment and cannot move forward without getting approval. Following training and evaluation, 28 pharmacists got the authorization to serve as internet hospital prescription review pharmacists.

Pharmacy Verification Supervision: Licensed pharmacists in retail pharmacies are required to examine transferred prescriptions prior to dispensing. A dual-way data synchronization mechanism between pharmacy enterprise resource planning systems and medical insurance inventory interfaces guarantees real-time precision of drug inventory data (8).

Post-Occurrence Quality Assurance Closed-Loop Regulatory Mechanism: A comprehensive-cycle supervision framework encompassing pre-occurrence, concurrent, and post-occurrence phases was established. Pre-event regulations regarding over-prescribing, over-expenditure, and contraindications are integrated at the hospital stage; real-time identity verification and smart medication supervision are utilized at retail drugstores; post-event supervision of diagnosis-based prescription, prescription circulation, and settlement is carried out at the administrative tier.

Data Monitoring and Assessment: Medical institutions routinely access electronic prescription implementation data through the smart medical insurance system to oversee the settlement situation. Unsuccessful transactions are monitored and examined. Statistical assessments of drug types, specifications, amounts, and fund outlays offer proof for policy enhancement.

Contingency Planning: Emergency procedures for medical insurance network disruptions and system malfunctions were formulated, along with improved cybersecurity and incident handling capabilities. Safeguarding of protected health-related information guarantees patient confidentiality and data safety.

### 2.4 Data collection and inclusion criteria

Data were gathered for 17,528 online accesses among patients making use of the electronic prescription circulation platform for medical-insurance-covered chronic disease refills during a 65-day span from January 23 to March 28, 2026. During the same period, 172,232 offline outpatient visits were registered at the same hospital.

Variables were classified into three domains: (1) demographic characteristics, including gender and age; (2) consultation behavior characteristics, including age, gender, and consultation time; and (3) prescription characteristics, including the number of drug types per prescription and drug categories.

### 2.5 Statistical analysis

Data were handled by using Microsoft Excel (version 16.59). All statistical computations were carried out with SPSS 25.0 software (IBM Corporation, Armonk, NY, USA). Continuous data were expressed as mean ± standard deviation (SD). Categorical data were presented as frequencies and percentages. Chi-square tests were employed for group comparisons of categorical variables, and independent-samples *t*-tests were utilized for continuous variables. To further explore the independent influencing factors of patients’usage behaviors, Generalized Estimating Equations (GEE) with a binary logistic link function were employed. Given that some patients contributed multiple visits (17,528 visits from 15,210 unique patients), an exchangeable working correlation matrix was specified with patient ID as the clustering variable to account for within-subject correlation. Age and gender were included as core confounding factors. Model fit and diagnostics were assessed using Variance Inflation Factors (VIF) to check for multicollinearity and the C-statistic (AUC) for discrimination. A two-sided *p*-value < 0.05 was regarded as statistically significant. For the multivariable analysis, the dependent variable, evening use, was operationally defined as a binary indicator where a consultation time falling between 18:00 and 22:00 was coded as 1, and 0 otherwise. This time window was selected to capture after-work utilization patterns. Regarding the covariate chronic disease medications, the variable was constructed to distinguish between medications for chronic diseases and those for non-chronic conditions. In the regression model, the reference category was set to chronic disease medications (coded as 1), while the comparison group represented non-chronic disease medications (coded as 0). This study was conducted over a 65-day period from January 23 to March 28, 2026, which encompassed the Spring Festival holiday. The Spring Festival period was defined as February 15 to 23, 2026 (Chinese New Year’s Eve to the seventh day of the Lunar New Year), consistent with the official 2026 Spring Festival holiday schedule in China. This period was coded as a binary interruption variable (1 for holiday dates, 0 otherwise) in the time-series analysis.

### 2.6 Ethical considerations

This study was approved by the Medical Research Ethics Committee of Xishan People’s Hospital of Wuxi City (No. 2026-K074-01). Informed consent was waived due to the retrospective nature of the study.

## 3 Results

### 3.1 Total volume of online and offline outpatient visits

**Figure 3:**
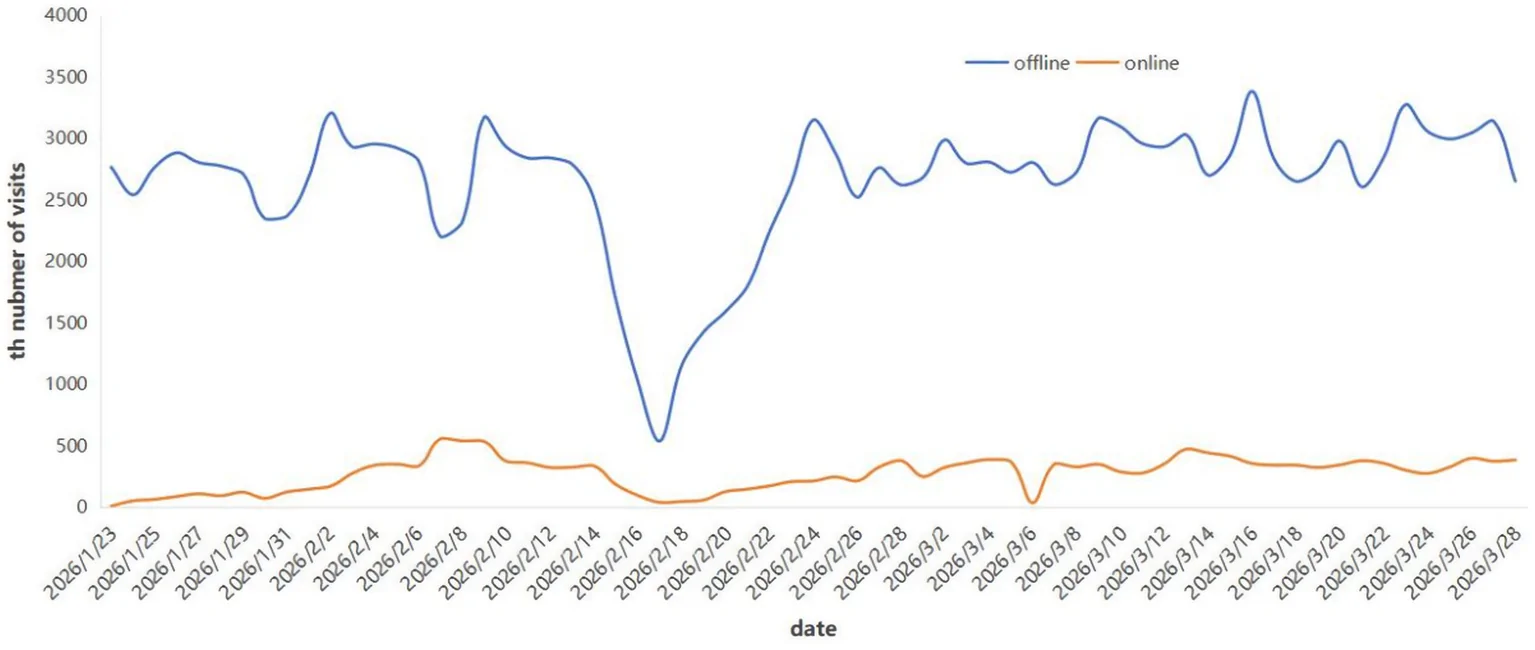
The number of online patients and offline outpatient patients based on the date.

During the 65-day research period, the hospital recorded 17,528 online visits for chronic disease medication refills, along with 172,232 offline outpatient visits. The daily average online visit volume was 270, while the average daily visit volume was 2,650. Online patients accounted for approximately 10.17% of offline patients. Affected by the Chinese New Year holiday (February 15 to 23, 2026), both online and offline visit volumes showed noticeable fluctuations during the observation period, as shown in Figure 3.

### 3.2 Demographic characteristics

A total of 17,528 online visits were incorporated into the final assessment, along with 172,232 offline outpatient visits. The majority of patients had only one visit, with a mean of 1.15 visits per patient. The baseline characteristics of the two patient groups are shown in Table 1.

**Table 1:** Descriptive characteristics of patients using online chronic medication refill service and general offline outpatient population.

Classification	Online		Offline	
	Number	*N* (%)	Number	*N* (%)
Total	17,528		172,232	
Gender				
Male	8,649	49.34	81,484	47.31
Female	8,879	50.66	90,748	52.69
Age (years)				
Mean (SD)	55.00 (13.57)		44.65 (20.95)	
Age group				
≤19	6	0.03	23,590	13.70
20–39	2,650	15.12	50,571	29.36
40–59	7,877	44.94	52,176	30.29
60–79	6,540	37.31	39,557	22.97
≥80	455	2.60	6,338	3.68

The online dataset (17,528 visits) constitutes a complete census of our Internet Hospital platform, predominantly serving chronic disease patients. The offline cohort, representing all outpatient visits (including acute care and pediatrics), is not directly comparable to the online group. No statistical tests were performed between groups due to inherent selection differences.

The online cohort was notably older than the general offline outpatient cohort. The mean age of online users was 55.00 years (SD = 13.57), compared to 44.65 years (SD = 20.95) for offline patients—a difference consistent with the comprehensive nature of general hospital outpatient services which encompass diverse medical needs including pediatrics and acute care.

This age disparity was further evident in the categorical distribution. The 20–39 age bracket comprised a smaller proportion of online users (15.12%) compared to the offline group (29.36%). Conversely, middle-aged and older adult patients (40–79 years) constituted the vast majority (82.25%) of the online service users, a significantly higher proportion than that observed in the offline group (53.26%). These discoveries verified that patients in the online and offline settings were separate groups, in line with the service orientation that online platforms mainly cater to patients in need of chronic disease medication refills.

### 3.3 Characteristics of consultation behaviors

Given that 84.85% of the online patients were 40 years old or above, the online patients were classified into two groups for behavioral analysis: those under 40 years (young group) and those 40 years or older (middle-aged and older group). Analysis of consultation time ranges within the online group showed different usage trends according to age (as shown in Table 2).

**Table 2:** Visit time patterns of online patients stratified by age (≤39 years vs. ≥40 years).

Classification	≤39 years old		≥40 years old		Effect size	*P*
	Number	*N* (%)	Number	*N* (%)		
Total	2,656	15.15	14,872	84.85		
Gender					*φ* = 0.043	<0.001
Male	1,177	44.31	7,472	50.24		
Female	1,479	55.69	7,400	49.76		
Visit time period					*V* = 0.165	<0.001
08:00–10:00	302	11.37	2,925	19.67		
10:00–12:00	361	13.59	2,471	16.62		
12:00–14:00	308	11.60	1,839	12.37		
14:00–16:00	360	13.55	2,296	15.44		
16:00–18:00	418	15.74	2,050	13.78		
18:00–20:00	620	23.34	2,563	17.23		
20:00–22:00	287	10.81	728	4.90		

Values are presented as No. (percentage). Chi-square test was used for group comparisons. *φ* (phi) and Cramér’s *V* were reported as measures of effect size. Two-sided *p* < 0.05 was considered statistically significant.

A notable disparity in the distribution of visit time intervals was found between patients aged 39 years or younger and those aged 40 years or older (Cramér’s *V* = 0.165, *p* < 0.001). The high-usage time for older patients (≥40 years) took place in the morning, from 08:00 to 10:00 (19.67% of this cohort). In comparison, the peak for younger patients (≤39 years) emerged in the evening, from 18:00 to 20:00 (23.34%). Moreover, patients who are younger had a likelihood more than two-fold to utilize the service in the late-evening time frame (20:00–22:00). Among them, 10.81% of their consultations took place within this period, while for older patients, it was merely 4.90%. These discoveries showed that online hospitals met the medication requirements of various populations beyond the regular outpatient time.

### 3.4 Characteristics of prescriptions

An analysis of the prescription data for the online group (17,528 visits, 16,682 prescriptions, 31,265 drugs) is presented in Table 3. The 846 visits (4.8%) without a corresponding prescription were excluded from prescription-level analysis.

**Table 3:** Prescription patterns and drug categories among online patients, stratified by age (≤39 years vs. ≥40 years).

Classification	≤39 years old		≥40 years old		Effect size	*p*
	Number	*N* (%)	Number	*N* (%)		
A. Polypharmacy patterns					*V* = 0.030	<0.001
Total number of prescriptions	2,496		14,186			
Number of medications per prescription						
1 type	1,354	54.25	7,116	50.16		
2–3 types	955	38.26	5,815	41.00		
More than 4 types	187	7.49	1,255	8.84		
B. Distribution of drug categories (%, column percentage)					*V* = 0.167	<0.001
Total number of drugs	4,498		26,767			
Drug category						
Cardiovascular system drugs	549	12.20	7,147	26.62		
Chinese patent medicines	1,137	25.27	4,942	18.41		
Digestive system drugs	543	12.07	2,534	9.44		
Hormones and endocrine-modulating drugs	223	4.96	2,179	8.12		
Antimicrobial agents	385	8.56	1,894	7.05		
Analgesic, antipyretic, anti-inflammatory, antirheumatic and antigout drugs	400	8.89	1,733	6.45		
Dermatological drugs	371	8.25	1,507	5.61		
Respiratory system drugs	220	4.89	1,004	3.74		
Urinary system drugs	72	1.60	885	3.30		
Hematological system drugs	81	1.80	727	2.71		
Ophthalmic drugs	181	4.02	599	2.23		
Nervous system drugs	67	1.49	550	2.05		
Otorhinolaryngological drugs	110	2.44	402	1.50		
Obstetric and gynecological drugs	47	1.04	179	0.67		
Vitamins and mineral supplements	28	0.62	182	0.68		
Anti-allergic agents	38	0.84	152	0.57		
Proctological drugs	19	0.42	134	0.50		
Immunomodulatory agents	15	0.33	34	0.13		
Water, electrolyte and acid–base balance regulators	4	0.09	27	0.10		
Stomatological drugs	7	0.16	18	0.07		
Psychotherapeutic drugs	0	0.00	16	0.06		
Antiparasitic agents	2	0.04	3	0.01		

Values are presented as No. (percentage). Drug category was conducted in accordance with the National Essential Medicines List (2018 Edition) issued by the National Health Commission of the People’s Republic of China. Chi-square test was used for all comparisons. Cramér’s *V* was reported as the effect size for polypharmacy patterns and drug category distribution. Two-sided *p* < 0.05 was considered statistically significant.

Although the pattern of polypharmacy (the quantity of medications per prescription) exhibited a statistically significant yet minimal-effect-size disparity between age groups (Cramér’s *V* = 0.030, *p* < 0.001), a notable difference was seen in medication categories. The distribution of medication types varied notably between the two age brackets (Cramér’s *V* = 0.167, *p* < 0.001), suggesting different clinical characteristics. For patients who are 40 years old or older, medications for the cardiovascular system were the most commonly prescribed category, making up 26.62% of all drugs for this patient group. In comparison, for patients whose age is ≤39 years, Chinese patent drugs were the most frequently-seen category (25.27%). In comparison with older patients, younger patients showed relatively higher percentages of prescriptions for drugs related to the digestive system, antimicrobial agents, and dermatological medications. This model indicated that online long-term medication refill services primarily handled cardiovascular and metabolic issues among older adults. In contrast, they served a wider range of acute and long-standing conditions, such as the utilization of traditional medicine, in younger adults.

### 3.5 Multivariable analysis of factors associated with evening use of online refills

To further explore the independent determinants of usage timing, a Generalized Estimating Equation (GEE) model was performed, with evening use set as the dependent variable. After adjusting for potential confounding factors and accounting for within-patient correlation using GEE, the model identified several significant predictors for evening visits (Table 4).

**Table 4:** Generalized Estimating Equations (GEE) analysis of factors associated with evening use of online chronic medication refill.

Variable	*B*	S.E.	Wald *χ*^2^	*p*	OR (95% CI)
Intercept	1.620	0.0893	328.826	<0.001	–
Gender (0 vs. 1)	0.125	0.0379	10.845	0.001	1.133 (1.052–1.221)
Age group (0 vs. 1)	−0.607	0.0482	158.787	<0.001	0.545 (0.496–0.599)
Weekend (0 vs. 1)	−0.036	0.0379	0.891	0.345	0.965 (0.896–1.039)
Spring Festival (0 vs. 1)	−0.395	0.0818	23.346	<0.001	0.674 (0.574–0.791)
Chronic disease medications (0 vs. 1)	−0.096	0.0389	6.041	0.014	0.908 (0.842–0.980)

Data were analyzed using Generalized Estimating Equations (GEE) with patient ID as the clustering variable to account for within-subject correlation from repeated visits. Reference groups: female, younger age group, weekday, non-Spring Festival period, non-chronic disease medications. Evening use was defined as consultation time between 18:00 and 22:00.

Gender was significantly associated with evening visits (Wald *χ*^2^ = 10.845, *p* = 0.001), with males showing a higher likelihood of visiting during evening hours (*B* = 0.125). Age group was the strongest predictor (Wald *χ*^2^ = 158.787, *p* < 0.001). Additionally, the Spring Festival period (Wald *χ*^2^ = 23.346, *p* < 0.001) and chronic disease medications (Wald *χ*^2^ = 6.041, *p* = 0.014) were significantly associated with the timing of visits. However, weekend status did not show a statistically significant association (Wald *χ*^2^ = 0.891, *p* = 0.345).

The final GEE model demonstrated good discrimination with an AUC of 0.768. Diagnostic tests revealed no evidence of multicollinearity, as all VIF values were close to 1 (Max VIF = 1.092).

## 4 Discussion

This real-world study described patient demographics, time-related utilization patterns, and prescription profiles linked to internet hospital-based external prescription circulation and chronic medication refill services in a district-level hospital located in eastern China. The present research results showed that this comprehensive model efficiently aimed at middle-aged and older patients having stable chronic diseases.

The model showed clear age-associated disparities in service time and medication needs, and it advanced digital health fairness by increasing access to vital medications outside of regular outpatient hours. These findings offer actionable proof to enhance telehealth work processes, improve chronic disease management approaches, and guide policy formulation for sustainable, patient-focused digital care provision.

In line with worldwide trends in telehealth utilization, the data of the present study verified that online chronic medication refill services served a demographically distinct patient group compared with traditional offline care (3). Online patients showed a notably higher age (average 55.00 compared to 44.65 years), where 84.85% were aged 40 years or older. This age-focused pattern was in line with prior research suggesting that older patients with chronic ailments are important beneficiaries of electronic prescription services, since they encounter more significant obstacles such as restricted mobility, transportation expenses, and infection hazards (6). By giving priority to the stable refill requirements for hypertension, diabetes, and cardiovascular ailments, this model directly tackled the deficiencies in long-term medication availability and decreased preventable hospital consultations (3, 4). Effect size analysis additionally made it clearer that age rather than gender was the driving factor for service utilization, strengthening the requirement for age-customized digital health design (6).

Nonetheless, the high adoption rate among odult adults needs to be understood within the wider framework of the digital gap (9–13). The notably reduced representation of young-aged adults (20–39 years) in the online refill group (15.12% vs. 29.36% offline) suggests that the digital divide might not a straightforward binary of use versus non-use, but rather a spectrum of engagement shaped by clinical need and service type. However, it is crucial to acknowledge that this interpretation is based solely on age and sex data. As previous studies demonstrated, factors such as device ownership, technology learning, and health status independently account for a substantial share of telehealth uptake among older adults (14–16). Given that our study lacked individual-level socioeconomic data, we cannot definitively conclude the drivers behind this observed spectrum of engagement. Therefore, this finding should be framed more cautiously as a hypothesis for future investigation rather than a confirmed conclusion. Future studies incorporating these variables are needed to validate the nature of this engagement spectrum and to better understand the specific barriers and facilitators for different demographic groups.

Analysis of temporal patterns uncovered remarkable age-related disparities in the timing of consultations. Older patients tended to prefer the morning hours (08:00–10:00), while younger patients showed a peak in the evening (18:00–20:00; Cramér’s *V* = 0.165; *p* < 0.001). This difference mirrored variations in daily schedules, job duties, and digital proficiency among different age brackets (3, 6). This kind of time-related flexibility is a key advantage of telehealth. It helps with medication adherence by fitting in with personal lifestyles and lessening obstacles associated with work timetables or mobility restrictions (17). For long-term health conditions such as high blood pressure and diabetes, continuous adherence is of great importance, and round-the-clock refill access overcomes a major logistical hurdle.

These results expand the current literature regarding telehealth utilization patterns and provide specific operational advice: staggered workforce scheduling, pharmacist reviews concentrated in the morning, and extended evening working hours can enhance efficiency and decrease waiting periods (17–19). From a health equity viewpoint, the availability of evening services allows individuals of working age to obtain care without having to skip work. Meanwhile, morning time slots are suitable for older users with earlier sleep–wake patterns, thus lessening age-based exclusion (6).

Prescription analysis highlighted clinically meaningful age-stratified medication profiles (Cramér’s *V* = 0.167; *p* < 0.001). Patients who are 40 years old or older mainly received cardiovascular medications, which is in line with the substantial burden of chronic non-communicable diseases within the middle-aged and older Chinese groups (18, 20). In comparison, younger patients placed greater reliance on Chinese patent medicines, digestive aids, and anti-inflammatory medications, which are related to acute or mild chronic situations. This disparity affirmed that efficient digital management of chronic diseases must adjust to diverse requirements throughout the lifespan. Although polypharmacy patterns differed statistically, the effect size was minimal (Cramér’s *V* = 0.030; *p* < 0.001), suggesting that the overall complexity of the regimen was comparable among different age groups. These outcomes verified the safety and suitability of online refills for managing stable chronic diseases and backed the incorporation of electronic prescription systems into regular primary care (3, 15).

To further elucidate the independent factors shaping these behaviors, a multivariable Generalized Estimating Equation (GEE) model was performed, which accounted for within-patient correlation from repeated visits. Gender (Wald *χ*^2^ = 10.845, *p* = 0.001), age group (Wald *χ*^2^ = 158.787, *p* < 0.001), the Spring Festival period (Wald *χ*^2^ = 23.346, *p* < 0.001), and chronic disease medications (Wald *χ*^2^ = 6.041, *p* = 0.014) emerged as significant independent predictors of evening use, whereas weekend status was not statistically significant (Wald *χ*^2^ = 0.891, *p* = 0.345).

Consistent with the descriptive statistics presented in Table 2, our adjusted GEE analysis confirms that younger age is a significant predictor of evening consultation preference. While younger patients (≤39 years) accounted for 34.15% of their total visits during evening hours compared to 22.13% for the older group (≥40 years), the multivariable model quantifies this relationship more precisely. After adjusting for covariates such as gender, weekend status, and chronic disease type, the odds of an older patient visiting in the evening were significantly lower than those of a younger patient (adjusted OR = 0.545, 95% CI: 0.496–0.599). This indicates that younger patients are nearly twice as likely to utilize evening slots as their older counterparts. This finding likely reflects differing daily schedules; younger individuals, who are more likely to be employed, may face conflicts with standard clinic hours and thus prefer evening appointments. In contrast, older patients, who include a higher proportion of retirees, have greater flexibility to seek care during the day. Our robust GEE model demonstrates a clear behavioral distinction: evening services primarily cater to the scheduling needs of the working-age population.

Additionally, male patients were found to be more inclined toward evening use in the GEE model, potentially due to more flexible daytime work arrangements. Furthermore, the increased evening utilization during the Spring Festival holiday likely reflects the limited availability of offline outpatient services and patients’ tendency to avoid hospital visits during traditional festivals. Notably, weekend status was not a significant predictor in the GEE model (*p* = 0.345), suggesting that the timing of visits is driven more by chronic disease management needs than by weekly work-rest cycles. Collectively, these findings deepen our understanding of the temporal preferences of internet hospital users and provide practical references for optimizing service scheduling for online chronic medication refills.

To further quantify the macro-level impact of the Spring Festival on overall service utilization—beyond its role as a covariate in the individual-level GEE model—we conducted a negative binomial interrupted time series analysis on daily prescription volume, adjusting for day-of-week effects and a linear time trend. The results revealed that prior to the holiday, daily prescription volume was on a significant upward trajectory (IRR = 1.137, 95% CI: 1.064–1.215, *p* < 0.001), indicating rapid growth in service adoption during the pre-holiday period. The onset of the Spring Festival was associated with a sharp immediate decline (IRR = 0.090, 95% CI: 0.023–0.354, *p* = 0.001), corresponding to a 91.0% reduction in daily volume. In the post-holiday period, although daily volumes fluctuated, the overall trajectory remained significantly below the pre-holiday secular trend (IRR = 0.897, 95% CI: 0.830–0.969, *p* = 0.006), suggesting that the holiday disrupted the growth trajectory without a clear rebound during the remaining observation window. Day-of-week effects were uniformly non-significant, confirming that the service maintained consistent utilization across the week. These findings corroborate the GEE results regarding the strong negative impact of the Spring Festival, while further revealing that this disruption was not merely a transient dip but rather a sustained interruption to the service‘s growth momentum—a pattern that warrants attention in service continuity planning during major national holidays.

The results of the current research present four practical operational and policy implications for promoting digital health equity and the quality of chronic care:

Age-segmented service design: We propose as untested design hypotheses that adopting streamlined interfaces, big fonts, and voice-based guidance for older adults could improve accessibility, an approach anchored in quantitative evidence on older adults’ telehealth preferences rather than solely inferred from our temporal usage data (6, 18, 19). Additionally, we hypothesize that increasing evening time slots would better serve patients of working age.

Targeted drug catalog management: Give priority to cardiovascular, endocrine, and antihypertensive drugs for the core older adult user group to enhance stock availability and dispensing efficiency (18, 20).

Temporal resource distribution: Clinical and pharmaceutical personnel deployment for online services should not adhere to a fixed 9-to-5 pattern. Resources (such as pharmacist review ability, clinician assistance) need to be adjusted to correspond to the predicted demand high-points: augmented in the morning (08:00–12:00) to handle the influx of older adults and in the evening (18:00–22:00) to meet the needs of younger users (3, 19).

Closed-loop quality control: Enhance real-time prescription assessment and medical insurance settlement surveillance to guarantee safety, compliance, and fraud deterrence (3, 15).

This study possesses multiple advantages, such as a substantial real-world sample, quantification of the effect size for group disparities, and in-depth description of behavioral and prescribing trends. Nonetheless, several limitations need to be recognized. First, this single-center retrospective design may restrict generalizability to other regions or hospital levels. Second, we utilized general outpatient data as a broad reference for offline care. However, due to the lack of specific coding for “chronic medication refills” in the offline system, we were unable to perform a direct comparative analysis between online and offline cohorts. Third, the current dataset only captures patient demographic and consultation utilization records, without extracting key operational process metrics embedded in insurance settlement data, including the proportion of prescriptions dispensed within the mandatory three-day collection period, pharmacist pre-review interception/failure rates, and the time turnaround from patient submission to prescription approval. No longitudinal follow-up data were collected to evaluate improvements in chronic medication refill continuity, limiting our ability to assess the system’s real-world operational efficiency and sustained medication access benefits. As synthesized by Xiao and Han, robust effectiveness evaluation of telehealth chronic disease management requires such process and outcome indicators, which our descriptive demographic findings alone cannot sufficiently support. Fourth, the study is subject to temporal constraints. The 65-day observation period encompassed the Chinese New Year holiday, during which travel and family traditions likely disrupted standard medication refill rhythms. Consequently, our findings may reflect festive or transitional patterns rather than the full-year norm, limiting generalizability to typical periods. Fifth, although Section 2.3 elaborates the dual computer-pharmacist prescription review quality control mechanism, we did not extract quantitative safety indicators such as prescription error interception rates stratified by the 23 online prescribers and 28 reviewing pharmacists. As Yang et al. illustrated, quantifiable metrics of prescribing errors and pharmacist interception rates are critical to empirically validate the performance of e-prescription safety supervision systems in Chinese hospitals; the absence of such numerical evidence weakens the evidentiary basis for our quality control claims. Additionally, the lack of individual-level socioeconomic data (e.g., income, education, device ownership and technology literacy) prevents definitive conclusions regarding the ‘digital divide’ or selection bias among older adults with limited digital proficiency. Future multi-center prospective investigations incorporating these variables are needed to validate the efficacy, safety, and cost–benefit aspects of integrated online-offline chronic disease management (21).

As suggested by Choi and Kim natural language processing combined with gradient boosting frameworks can meaningfully enhance prediction over conventional covariate sets in public health contexts (14). While the present analysis—constrained by the structured nature of our dataset—serves as a foundational descriptive step, future studies with access to free-text consultation records, unstructured diagnosis text, or longitudinal behavioral data could apply these methods to achieve more granular user segmentation and prediction of usage timing. Such approaches would also help address the modest discriminative performance observed in our current model, as richer predictors would likely capture more of the complexity underlying healthcare-seeking behavior.

## 5 Conclusion

Internet hospital-centered external prescription and long-term medication refill services present a strong, equity-promoting model that consistently backs middle-aged and older adult patients suffering from chronic diseases. Differences related to age in terms of timing, behavior, and medication need call for personalized and stratified digital health approaches instead of a uniform design. The practical-world data offer actionable understandings to optimize the allocation of resources, enhance chronic disease management, reinforce medication safety, and direct health policy for combined online-offline care. Scaling up this model is able to broaden fair access to vital medications, relieve the pressure on offline healthcare systems, and enhance population health during the accelerating digital health transformation.

## Data Availability

The raw data supporting the conclusions of this article will be made available by the authors, without undue reservation.
